# Neutrophils Require Activation to Express Functional Cell-Surface Complement Receptor Immunoglobulin

**DOI:** 10.3389/fimmu.2022.840510

**Published:** 2022-03-04

**Authors:** Annabelle G. Small, Khalida Perveen, Trishni Putty, Nikita Patel, Patrick Quinn, Mihir D. Wechalekar, Charles S. Hii, Alex Quach, Antonio Ferrante

**Affiliations:** ^1^ Department of Immunopathology, South Australia (SA) Pathology, Women’s and Children’s Hospital, North Adelaide, SA, Australia; ^2^ Department of Molecular and Biomedical Science, School of Biological Sciences, University of Adelaide, North Adelaide, SA, Australia; ^3^ Robinson Research Institute and Adelaide Medical School, University of Adelaide, Adelaide, SA, Australia; ^4^ Rheumatology Department, College of Medicine and Public Health, Flinders Medical Centre, Flinders University, Bedfort Park, SA, Australia; ^5^ Department of Allergy and Immunology, Women’s and Children’s Health Network, North Adelaide, SA, Australia

**Keywords:** neutrophil, CRIg/VSIG4, complement receptors, inflammatory mediators, intracellular signaling, microbial killing, cytokines, neutrophil extracellular trap

## Abstract

The phagocytosis-promoting complement receptor, Complement Receptor Immunoglobulin (CRIg), is exclusively expressed on macrophages. It has been demonstrated that expression in macrophages could be modulated by inflammatory mediators, including cytokines. This raised the possibility that a major phagocyte, the neutrophil, may also express CRIg following activation with inflammatory mediators. Here we show that resting peripheral blood neutrophil lysates subjected to protein analysis by Western blot revealed a 35 kDa CRIg isoform, consistent with the expression of CRIg mRNA by RT-PCR. By flow cytometry, CRIg was detected intracellularly and in very minor amounts on the cell surface. Interestingly, expression on the cell surface was significantly increased to functional levels after activation with inflammatory mediators/neutrophil activators; N-Formylmethionine-leucyl-phenylalanine, tumor necrosis factor (TNF), Granulocyte-Macrophage Colony stimulating Factor (GM-CSF), bacterial lipopolysaccharide, leukotriene B4 and phorbol myristate acetate. The increase in expression required p38 MAP kinase and protein kinase C activation, as well as intracellular calcium. Neutrophils which were defective in actin microfilament reorganization due to a mutation in ARPC1B or inhibition of its upstream regulator, Rac2 lose their ability to upregulate CRIg expression. Inhibition of another small GTPase, Rab27a, with pharmacological inhibitors prevented the increase in CRIg expression, suggesting a requirement for the actin cytoskeleton and exocytosis. Engagement of CRIg on TNF-primed neutrophils with an anti-CRIg monoclonal antibody increased the release of superoxide and promoted the activation of p38 but not ERK1/ERK2 or JNK MAP kinases. The TNF-induced increase in killing of *Staphylococcus aureus* was blocked by the anti-CRIg antibody. Adding to the anti-microbial role of CRIg, it was found that GM-CSF priming lead to the release of neutrophil extracellular traps. Interestingly in contrast to the above mediators the anti-inflammatory cytokine IL-10 caused a decrease in basal expression and GM-CSF induced increase in CRIg expression. The data demonstrate that neutrophils also express CRIg which is regulated by inflammatory mediators and cytokines. The findings show that the neutrophil antimicrobial function involving CRIg requires priming as a means of arming the cell strategically with microbial invasion of tissues and the bloodstream.

## Introduction

CRIg or V-set and immunoglobulin domain–containing 4 (VSIG4) has distinct structural and biological properties from the classical complement receptors, CR3 and CR4 (1, 2). Its role in innate immunity is exemplified by its ability to promote the rapid clearance of blood-borne bacteria in both a complement-dependent and -independent pattern recognition receptor manner (3, 4). CRIg has also been found to suppress the immune response (5, 6) and inhibit the activation of the alternative pathway (7, 8). These properties have further led to the findings that CRIg is protective in a number of autoimmune/chronic inflammation disease models (7, 8). The restriction of CRIg expression to macrophages, and in particular to subpopulations of fixed tissue macrophages such as the liver Kupffer cells in the mouse, has implicated the importance of CRIg-expressing macrophages in immunity against infection and protection against inflammatory diseases (1, 3–5, 8). However, recent reports have shown that CRIg protein is undetectable in human liver tissue, suggesting that its role in immunity in humans still remains to be defined (9). Our previous work examining CRIg on human monocyte-derived macrophages demonstrated that CRIg expression could be substantially depressed as well as increased by treating macrophages with inflammatory mediators and drugs such as dexamethasone (10, 11). This raised the possibility that other phagocytes such as neutrophils may express CRIg following activation by inflammatory mediators and cytokines, and was the subject of this study.

We now report that human neutrophils also express CRIg protein, but it is only significantly expressed on the cell surface following activation by endogenous and exogenous inflammatory mediators, in a regulatory manner since IL-10 was also able to down regulate its expression. Engaging CRIg on the neutrophil surface leads to a significant activation of the key functional response of the oxygen-dependent respiratory burst and associated intracellular signaling molecule activation, p38 and bacterial killing. The ability to increase expression on the cell surface was dependent on p38, PKC, intracellular Ca^2+^ and small GTP binding proteins and the cytoskeleton. This identifies a unique process of regulating CRIg expression and arming neutrophils at sites of infection and inflammation.

## Materials And Methods

### Ethical Statement

The procurement of human blood and all experimental procedures were approved by the Human Research Ethics Committee of the Women’s and Children’s Health Network (WCHN), Adelaide, South Australia, and the Southern Adelaide Clinical Human Research Ethics Committee in accordance with The National Statement on Ethical Conduct in Human Research (2007, updated 2018; National Health and Medical Research Council Act 1992). Blood was collected from healthy volunteers by venipuncture with their informed consent. Results from studies on ARPC1B-deficient neutrophils were obtained as part of the clinical laboratory assessment of the patient and informed consent was obtain from the parents to publish the results.

### Reagents

RPMI 1640 tissue culture medium, fetal calf serum (FCS) and L-glutamine were purchased from SAFC Biosciences (Lenexa, Kansas). Recombinant human tumor necrosis factor (TNF), granulocyte-macrophage colony stimulating factor (GM-CSF), and interleukin-10 (IL-10) were purchased from ProSpec-Tany Technogene (Rehovot, Israel). Phorbol myristate acetate (PMA), lipopolysaccharide (LPS) from *Escherichia coli* O127:B8, N-Formyl-Met-Leu-Phe (fMLF), wortmannin, BAPTA-AM (calcium chelator), and Nexinhib20 (Rab27 inhibitor) were purchased from Sigma-Aldrich (St. Louis, MO). Leukotriene B4 (LTB4) was purchased from Cayman Chemical (Ann Arbor, MI). SB202190 and SB203580 from SelleckChem (Houston, TX). The PKC inhibitor GF109203X was purchased from Biomol Research Laboratories (Plymouth Meeting, PA). Rac-1 inhibitor, NSC 23766, and the Rac-1/Rac-2 inhibitor, EHT 1864, were purchased from TOCRIS (Bristol, UK).

### Antibodies

FITC-conjugated goat anti-rat antibody and, unlabeled and PE-conjugated mouse anti-human CRIg monoclonal antibody (clone 6H8, 1:200 for Western blotting), were from Santa Cruz Biotechnology (Dallas, TX). Fluorochrome-conjugated antibodies to CD45 (APC-H7; clone 2D1), CD11b (PE; clone D12), and mouse IgG1-κ isotype control (PE; clone MOPC-21), were from BD Biosciences (Franklin Lakes, NJ). The rabbit anti-ARPC1B antibody (HPA004832, 1:1000) and mouse monoclonal anti-GAPDH (clone 71.1, 1:20,000) were from Sigma-Aldrich. The polyclonal HRP-conjugated rabbit anti-mouse (P0260), anti-goat (P0449), and goat anti-rabbit (P0448) immunoglobulin antibodies (1:2000) were purchased from Dako (Glostrup, Denmark). The polyclonal anti-myeloperoxidase (MPO) antibody was purchased from Abcam (Cambridge, UK). The Alexa Fluor 488-conjugated goat anti-rabbit IgG (H+L) highly cross-adsorbed secondary antibody was purchased from Invitrogen (Waltham, MA).

### Purification of Neutrophils

Neutrophils were isolated from the blood of healthy donors using the rapid-single-step method, as described previously (12). Briefly blood was layered on Hypaque-Ficoll, d = 1.114 and centrifuged at 600 × g for 35 min. The bottom leukocytes band containing neutrophils was harvested, washed and used immediately in these experiments. Viability was >99% and purity of the order of 98%.

### Reverse Transcription PCR Assays

In brief, total RNA was extracted from freshly isolated neutrophils using TRIzol reagent (Invitrogen, Waltham, MA). cDNA was prepared using 300 ng RNA using iScript™ cDNA synthesis kit (Bio-Rad, Hercules, CA). RT-PCR analysis was performed using AmpliTaq Gold^®^ 360 Master Mix (Applied Biosystems, Waltham, MA) with the following conditions: initial denaturation for 5 min at 95°C followed by 40 cycles at 95°C for 30 s, 60°C for 30 s and 72°C for 30 s using a Bio-Rad MyCycler. The primer pairs used were for human CRIg (Forward: 5’-ACACTTATGGCCGTCCCAT-3’; Reverse: 5’-TGTACCAGCCACTTCACCAA-3’) and GAPDH (11). The PCR products were visualized by 2% agarose gel electrophoresis alongside 1 kb Plus DNA ladder (Invitrogen).

### Sanger Sequencing

Genomic DNA was isolated from heparinized blood using the Flexigene DNA kit (Qiagen, Hilden, Germany). *ARPC1B* gene exon 2 and the flanking intronic regions were amplified using AmpliTaq Gold^®^ 360 Master Mix (Applied Biosystems) with 0.1 μM of each M13-tagged primer (Forward: GCTGCCCCTCTAAACTGAGG; Reverse: AACTTTAACCCAGGAGGCCC), and 25–50 ng DNA in a 25 μL final PCR volume. Thermal cycling conditions were an initial denaturation of 95°C for 10 min, followed by 35 cycles of 95°C for 30 s, 60°C for 30 s, and 72°C for 60 s; with a final extension at 72°C for 7 min. The PCR products were purified using Illustra ExoProStar1-Step (GE HealthCare, Chicago, IL) and sequenced using BigDye Terminator v3.1 on an ABI 3730 DNA Analyzer (Applied Biosystems). Mutation detection was performed by alignment with reference sequence LRG_1188 using Mutation Surveyor v4.0.11 (SoftGenetics, State College, PA).

### Western Blot

Neutrophil pellets were resuspended in 100 µL of lysis buffer containing 20 mM HEPES, pH 7.4, 0.5% Nonidet P-40 (v/v), 100 mM NaCl, 1 mM EDTA, 2 mM Na_3_VO_4_, 2 mM dithiothreitol, 1 mM PMSF and 1 µg/mL of each protease inhibitor (benzamidine, leupeptin, pepstatin A, and phenylmethylsulfonyl fluoride (PMSF), from Sigma-Aldrich, and aprotinin from Calbiochem (Merck, Darmstadt, Germany). Total protein in the soluble fractions were quantitated using the Qubit™ Protein Assay Kit on a Qubit 3.0 (Invitrogen). After addition of Laemmli buffer, the samples were boiled at 100°C for 5 min and subjected to 10% SDS-PAGE at 170 V for approximately 1 hour, using the Mini-PROTEAN 3 system (Bio-Rad), then transferred to nitrocellulose membranes using the Trans-Blot^®^ Turbo™ Transfer System (Bio-Rad) and examined by 0.1% Ponceau S staining. After blocking in TBST with 5% skim milk (blocking solution) for 1 h, the membrane was incubated with the anti-CRIg (6H8) primary antibodies in blocking solution for 1 h at RT. The membrane was washed in blocking solution and then incubated with secondary HRP-conjugated antibodies in blocking solution for 1 h at RT. Immunoreactive material was detected using the Western Lightning Plus-ECL Enhanced Chemiluminescence Substrate (PerkinElmer, Waltham, MA), with protein bands visualized on a ChemiDoc™ XRS+ Imager and quantitated using Image Lab™ Software, Version 3.0 (Bio-Rad).

In studies examining the activation of p38, JNK and ERK1/2, purified neutrophils at 2 x 10^6^ cells/mL in HBSS (without serum) were stimulated with TNF (10^3^ U/mL) for 20 min at 37°C/5%CO_2_ air. Then anti-CRIg antibody (6H8) was added at 4 μg/mL for 15 min at 37°C/5%CO_2_. The reaction was stopped by adding cold HBSS. The cells were pelleted by centrifugation at 600g for 5 min at 4°C and cell lysates prepared for western blot. For detection of phospho-protein, rabbit polyclonal anti-p-p38 (Thr180/tyr182)-R (sc-17852-R), mouse monoclonal anti-p-JNK antibody (Thr 183/Tyr 185) (G-7; sc-6254), and mouse monoclonal anti-p-ERK 1/2 antibody (pT202/pY204.22A) (sc-136521) primary antibodies were used at 1:2000 dilution in 5% BSA for 1 h at room temperature or overnight at 4°C followed by appropriate secondary antibodies. To detect total amount of protein, rabbit polyclonal anti-p38α antibody (C-20: sc-535), rabbit polyclonal anti-JNK1/3 antibody (C-17: sc-474), rabbit polyclonal anti-ERK2 (C-14); sc-154) (1:2000) or mouse anti-GAPDH monoclonal antibody (1:10000) were used after blocking with skim milk (blocking solution) and then the appropriate secondary antibodies added. These were developed as described above.

### Flow Cytometry

Cell surface expression of CRIg, CD45, and CD11b was analysed by flow cytometry as previously described (10, 11). Briefly, 2.5 × 10^5^ cells were incubated with 100 μg human IgG (Kiovig, Baxter, Old Toongabbie, NSW, Australia) for 15 min and treated with fluorochrome-conjugated anti-human primary antibodies. Cells were washed after 20 min incubation. For experiments requiring intracellular staining, surface antigens were stained as above before fixation and permeabilization with the BD Fixation/Permeabilization kit (BD Biosciences) as per the manufacturer’s instructions. Intracellular antigens were immunostained in the presence of BD Perm/Wash™ Buffer to maintain permeabilization for 20 min in the dark on ice. Following staining, cells were washed twice in 2 mL of BD Perm/Wash™ Buffer. Cells were examined by flow cytometry using a BD FACSCanto I and data analysed using FlowJo 10.1 (FlowJo, LLC, Ashland, Oregon).

### Examination of CRIg Expression by Confocal Microscopy

For imaging, neutrophils were treated in the same manner as for the flow cytometry studies with some differences. Briefly, cells were either left untreated or treated with PMA (100 nM) for 15 min at 37°C/5%CO_2_. The reaction was stopped by the addition of ice-cold HBSS and cells centrifuged. The cells were resuspended in PE-conjugated anti-CRIg antibody (6H8) and incubated for 30 min at 4°C. Five minutes prior to end of incubation, DAPI nuclear stain was added. Cells were transferred to Superfrost^®^ microscope slides (Thermo Scientific, Waltham, MA) by using the Cytospin 3 centrifuge (Shandon Scientific, Cheshire, UK). Cells were air dried and mounted with a fluorescent mounting medium (Dako). The samples were visualized under an Olympus FV3000 Confocal Microscope at 600× magnification with oil at the Adelaide Microscopy’s facility (The University of Adelaide, South Australia) and the images were acquired by using FV31S-SW Image software tools. The images were processed by using FIJI image software (13).

### Chemiluminescence Assay

Lucigenin-dependent chemiluminescence assay was performed as previously described (14). Briefly, 1 x 10^6^ neutrophils were added to 125 µg lucigenin (bis-N-methylacridinium nitrate, Sigma-Aldrich) in 500 µL HBSS. Chemiluminescence was measured using a LB 953 Autolumat Plus luminometer (Berthold Technologies, Bad Wildbad, Germany), and peak fluorescence recorded. Data is expressed as mean relative luminescence units (RLU).

### Bactericidal Assay

Neutrophil bactericidal activity was assayed similarly to that previously described (12). Briefly, 1 × 10^6^ neutrophils were incubated with or without TNF (10^3^ U/mL) for 20 min at 37°C in 400 µL HBSS supplemented with 10% human AB serum. Anti-CRIg was added at 2 µg/mL, as stipulated in the results, and following a 2 min incubation, this was followed by the addition of 1 × 10^6^ *S. aureus* (NCTC strain 6571, London, UK) to a final reaction volume of 500 µL in 5 mL polypropylene round bottom screw top tubes. The tubes were gassed with 5% CO_2_ and incubated on a rocking platform for 60 min at 37°C. Samples were taken from the tubes at 0 and 60 min, and diluted with sterile distilled water (1:200). Fifty µL of each dilution were spread on horse blood agar plates, with colonies counted following overnight incubation at 37°C.

### Neutrophil Extracellular Traps Assay

NET formation by neutrophils was examined using Propidium iodide (PI) and anti-MPO antibody by flow cytometry and microscopy. Briefly, neutrophils at 2 × 10^6^ (HBSS/2% human AB serum) were either left untreated or treated with GM-CSF (25 ng/mL) for 20 min, followed by overnight treatment with or without anti-CRIg (4 µg/mL) at 37°C/5%CO_2_. Cells were immunostained with rabbit polyclonal anti-MPO antibody at 1:150 dilution for 30 min. Following washing in PBS/2% FCS, Alexa Fluor 488-conjugated goat anti-rabbit IgG (H+L) was added (1 µg/mL) for a further 30 min. Following a final wash, propidium iodide (5 µL, 3.75 mM solution from L7010 LIVE/DEAD™ Cell-Mediated Cytotoxicity Kit, Invitrogen) was added for a further 10 min, followed by (1) for flow cytometry, immediately acquisition on a BD FACSCanto I; or (2) for fluorescent imaging, air-drying of slides, then overnight mounting with ProLong™ Gold Antifade Mountant (Invitrogen), with images acquired on an Olympus BX51 Fluorescence Microscope at 40x and analysis using FIJI image software.

### Statistical Analysis

GraphPad Prism 8.0 (GraphPad Software, San Diego, CA) was used for statistical analysis. Mean differences were compared using t-tests (for comparisons of two groups) or one-way ANOVA followed by multiple-comparison tests (for comparisons of three of more groups). *P* values < 0.05 were considered statistically significant.

## Results

### CRIg Expression in Neutrophils

**Figure 1:**
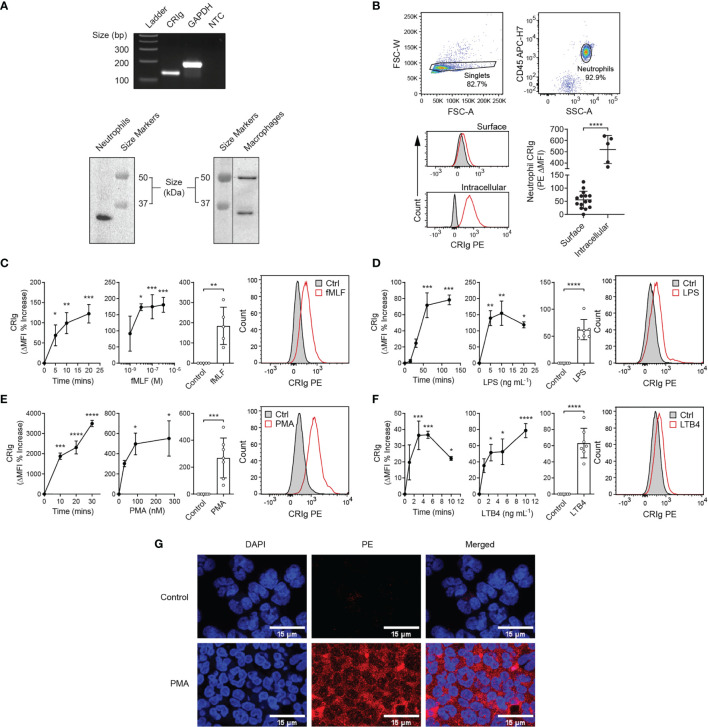
Expression of CRIg by human neutrophils. **(A)** Representative gel of CRIg (*VSIG4*) and *GAPDH* cDNA amplicons from neutrophil RNA (top) and Western blot of neutrophil (lower left) and MDM (lower right) lysates stained using anti-CRIg (clone 6H8) monoclonal antibody. NTC, no template control. **(B)** Surface and intracellular CRIg expression in neutrophils, including gating strategy with histogram overlays of PE anti-CRIg antibody (red line) and isotype control (black line with grey shading) staining. Bottom graph shows MFI of CRIg PE minus isotype control (ΔMFI) for surface (*n* = 15 individuals) and intracellular (*n* = 5 individuals) staining. Data are presented as individual values and as mean ± SD. Two-tailed, unpaired t-test with Welch’s correction compared with surface expression. One-way analysis of variance (ANOVA) with Tukey’s post-test compared between all sample types. **(C–F)** CRIg expression on activated neutrophils. Upper panels show changes in CRIg expression on the surface of neutrophils over time in response to fMLF (10^-6^ M), LPS (10 ng/mL), PMA (100 nM), and LTB4 (5 ng/mL). Middle panels show representative histograms. Lower panels show agonist concentration related effect at 20 min (fMLF), 60 min (LPS), 20 min (PMA), and 5 min (LTB4). Expression was calculated as ΔMFI and then these were expressed as a percentage increase over control treatment (*n* = 3 experiments). Summary of the stimulation of neutrophil CRIg expression by fMLF, LPS, PMA and LTB4 at optimal times (20, 60, 20, and 5 min, respectively) and concentrations (10^-6^ M, 10 ng/mL, 100 nM and 5 ng/mL, respectively). Measurements were taken from distinct specimens and graphs show mean ± SD of these experiments. **(G)** Shows CRIg expression on PMA (100 nM)-activated neutrophils by confocal microscopy. DAPI has been used to stain the cell nucleus to locate cells and PE labelled anti-CRIg for CRIg expression. Statistical analyses **(C–F)**, One-way ANOVA with Dunnett’s post-test compared with expression at the start of the time-course or in the absence of added inflammatory mediator. **(G)** Kruskal-Wallis test with uncorrected Dunn’s post-test compared with control treatment. Significance levels are indicated by asterisks: *P < 0.05, **P < 0.01, ***P < 0.001, ****P < 0.0001.

Here, we report that human neutrophils express CRIg (**Figure 1**). Neutrophils purified from blood of healthy donors express CRIg mRNA by RT-PCR (**Figure 1A**, upper panel), and protein by Western blot using the anti-CRIg monoclonal antibody, 6H8 (**Figure 1A**, lower left panel). One isoform of CRIg was detected in neutrophil lysates, migrating at ~35 kDa. In human monocyte derived macrophages (MDM) the antibody also detected CRIg at 35 kDa, but in addition showed a longer form at ~50 kDa (**Figure 1A**, lower right panel) (1). Examination of the resting neutrophil cell surface shows very low expression in comparison to intracellular levels, revealed by flow cytometry using the same anti-CRIg monoclonal antibody (**Figure 1B**).

### Activated Neutrophils Express Functional Cell Surface CRIg

Further investigations revealed that activating neutrophils with inflammatory mediators, fMLF, LPS, PMA or LTB4 caused a significant increase in surface CRIg expression (**Figures 1C** **–F**), shown by flow cytometry analysis. **Figure 1G** shows the increased expression of CRIg on the neutrophil surface induced by PMA viewed by confocal microscopy. These effects occurred in a concentration dependent manner (**Figures 1C** **–F**). The concentration related effects of fMLF were consistent with previously established findings (15) that low concentrations of fMLF promoted chemotaxis and higher concentration activated the cell to release superoxide and granule constituents. The effects seen with the other agonists were also consistent with the concentrations that cause the activation of other neutrophil responses. Monitoring the time-related changes in expression, showed that the response was rapid. The agonists apart from LPS caused a significant increase within the first 5 min of incubation.

**Figure 2:**
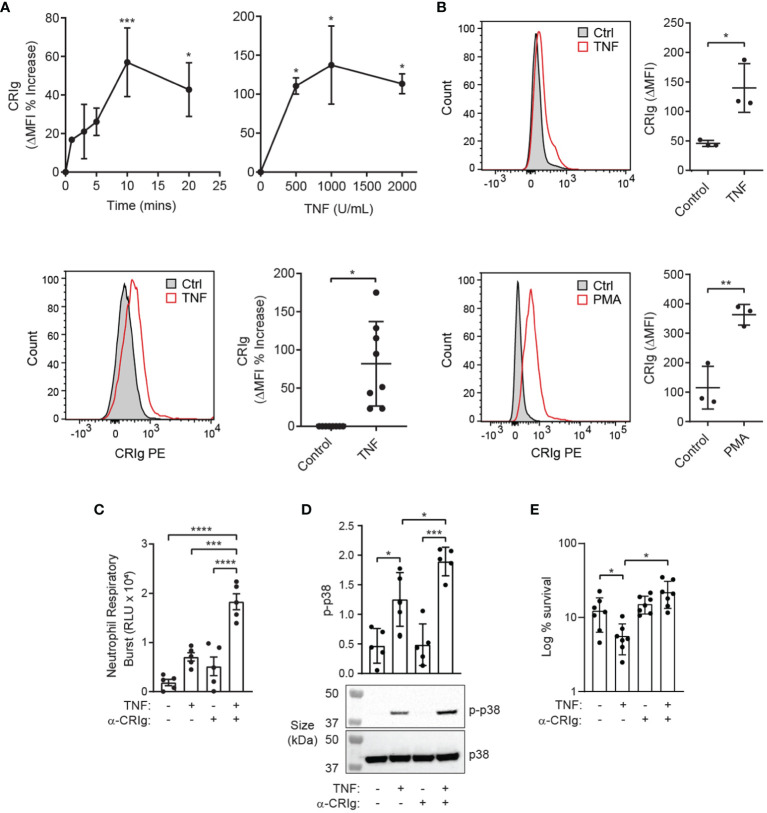
TNF induces functional CRIg expression on neutrophils. **(A)** Shows CRIg expression changes on the surface of neutrophils over time-courses (10^3^ U/mL) and TNF concentration ranges (20 min), along with representative histograms (lower left panel) of responses to TNF. Expression was assessed as described in **Figure 1** with ΔMFI as a percentage increase over control treatment. Cumulative data from different runs of the stimulation of neutrophil CRIg expression by TNF at optimal time of 20 min and concentration of 10^3^ U/mL is shown in the lower right panel. **(B)** Neutrophil surface CRIg expression in whole blood treated with 10^4^ U/mL TNF or 100 nM PMA for 20 min. **(C)** Neutrophils were treated for 20 min with 10^3^ U/mL TNF with or without challenge with 4 µg/mL anti-CRIg mAb (6H8), and the respiratory burst measured by lucigenin-induced chemiluminescence (RLU, relative luminescence units). **(D)** Examination of activation of p38 in neutrophils pre-treated with TNF and then stimulated with the anti-CRIg monoclonal antibody, assessed by western blot using anti-phosphorylated p38 (p-p38) and anti-p38 specific monoclonal antibodies. **(E)** Anti-CRIg antibody blocks the TNF-induced priming for *S. aureus* killing. Data is expressed as mean ± SD of seven experiments, expressed as log10% bacterial survival. Data is shown as individual measurements and as mean ± SD of at these measurements. The lower panels show a representative Western blot. Statistical analyses: **(A)**, One-way ANOVA with Dunnett’s post-test compared with expression at the start of the time-course or in the absence of added inflammatory mediator. **(B)**, Two-tailed, unpaired t-test compared with control expression; **(C–E)**, One-way ANOVA with Tukey’s post-test compared between all treatments. Significance levels are indicated by asterisks: *P < 0.05, **P < 0.01, ***P < 0.001, ****P < 0.0001.

TNF is known to prime neutrophils for increased anti-bacterial killing (12). Here, we show that TNF increases the expression of cell surface CRIg, rapidly (**Figure 2A**). This suggests that TNF promotes a rapid increase in CRIg surface expression, possibly through a translocation of the protein to the cell surface. Adding either TNF or PMA to whole blood also caused an increase of surface CRIg expression on neutrophils (**Figure 2B**).

**Figure 3:**
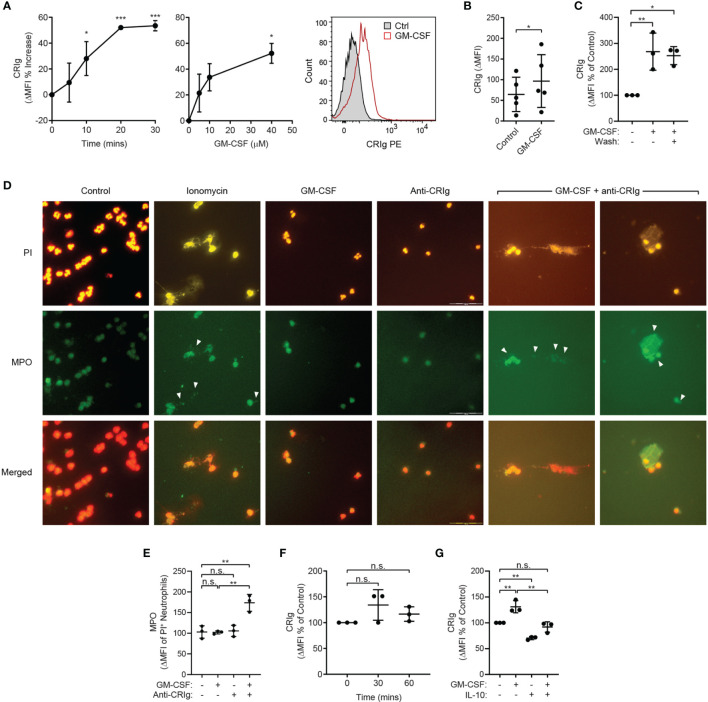
Effect of GM-CSF on CRIg expression and CRIg-induced release of NETs and regulation by IL-10. **(A)** CRIg expression on activated neutrophils. Data show changes in CRIg expression by flow cytometry on the surface of neutrophils over time in response to GM-CSF (40 ng/mL) and the cytokine concentration related effect at 30 min. Expression was calculated as ΔMFI and then these were expressed as a percentage increase over control treatment (*n* = 3 experiments). **(B)** Summary of the stimulation of neutrophil CRIg expression by GM-CSF for 30 min incubation and a concentration 40 ng/mL. Measurements were taken from distinct specimens and graphs show mean ± SD of these experiments. **(C)** Shows the effect of washing (Wash) the cells after priming with GM-CSF. Neutrophils were treated for 10 min with 40 ng/mL GM-CSF and then either washed or left intact. After a further 10 min incubation the level of surface CRIg was assessed by flow cytometry. **(D)** examination of CRIg induced NET release. Photomicrographs show the release of NETs caused by anti-CRIg antibody only after priming with GM-CSF, with no effect seen by treating neutrophils with GM-CSF or anti-CRIg antibody alone. Shown is the PI staining, MPO staining using indirect immunofluorescence and the combination of staining for DNA and MPO. In these investigations the neutrophils were treated with 25 ng/mL of GM-CSF for 20 min and then with anti-CRIg antibody. The cells were examined after a further 18 h incubation. Scale is represented by lines in the photo. **(E)** the NETs were semi-quantitated by flow cytometry. Data shows the MPO expression on PI labelled cells. **(F)** Shows the effects of neutrophil priming by plasma coated surfaces on CRIg expression over 60 min incubation. Data are presented as individual values and as mean ± SD of these values/experiments. **(G)** Shows the effects of IL-10 (100 ng/mL) on basal and GM-CSF (40 ng/mL) on CRIg expression in neutrophils. One-way ANOVA with Dunnett’s **(A, C, G)** or Sidak’s **(E, F)** post-test compared between all treatments and **(B)** Two-tailed, unpaired t-test compared with control expression. Significance levels are indicated by asterisks: *P < 0.05, **P < 0.01, ***P < 0.001, n.s., not significant.

To determine whether engagement of CRIg expressed on the surface of human neutrophils is capable of eliciting an immune response, the TNF-primed neutrophils were treated with an anti-CRIg monoclonal antibody (clone 6H8, Santa Cruz Biotechnology). Engagement of CRIg led to a significant stimulation of superoxide production, measured by the lucigenin-dependent chemiluminescence assay (**Figure 2C**) (14). These results show that CRIg not only promotes the phagocytosis of bacteria (1), but also elicits an associated NADPH oxidase activation involved in the bactericidal activity. The functional activity of CRIg was further gauged by examining the activation of intracellular signals, MAP kinases, involved in a range of neutrophil functions (13, 14). Neutrophils were pre-treated with TNF to increase the level of surface CRIg expression and then they were treated with the anti-CRIg monoclonal antibody. Examination of the activation of the MAP kinases showed that TNF activated p38 (**Figure 2D**) but not ERK or JNK (unpublished observations), as previously demonstrated (16). Neutrophils treated with the anti-CRIg antibody alone did not show activation of any of these MAP kinases (**Figure 2D**, unpublished data), But when primed with TNF the cells showed an increase in p38 activation in response to the anti-CRIg antibody but not ERK and JNK (data not presented), suggesting that neutrophil activation *via* CRIg engagement does not require these two MAP kinases. The bactericidal promoting properties of CRIg in TNF primed neutrophils were further developed by showing that the TNF-induced increase in killing of *S. aureus* was abrogated by adding the anti-CRIg antibody (**Figure 2E**). This work was extended to another cytokine which is well known for its neutrophil priming properties, GM-CSF (17). The data presented in **Figures 3A** **, B** show that that GM-CSF treated neutrophils display an increased expression of CRIg on their cell surface, in a time and concentration related manner. Similar to the effects of other inflammatory mediators this cytokine showed effects within the 20 min of incubation. We examined whether the priming required the continuous presence of the cytokine. Treating neutrophils with GM-CSF for 15 min and then washing the cells to remove exogenous GM-CSF, maintained the increase in CRIg expression (**Figure 3C**). An important aspect of neutrophil anti-microbial function is the formation of extracellular traps (NET), projections which characteristically are composed of extracellular DNA and granule proteins such as myeloperoxidase. Thus, to increase the significance of our findings we examined whether engagement of CRIg promoted the release of NETs. In these studies, we used GM-CSF as a priming agent since this has been shown to increase the expression of CRIg (**Figure 3**), but not the release of NETs (18). To view the release of NETs, the DNA dye PI and granule constituent, MPO, were measured in treated cells, both microscopically and by flow cytometry. The data presented in **Figure 3D** show images of cells treated with GM-CSF, anti-CRIg antibody and a combination of these two. While neither of the agonists had an effect, neutrophils primed with GM-CSF responded to the anti-CRIg by the release of NETs. When cells were gated for both expression of DNA and MPO, no NETs were released with either treatment with GM-CSF or Anti-CRIg antibody. However, there was a significant increase in NETs when cells were primed with GM-CSF and then challenged with anti-CRIg antibody (**Figure 3E**). Finally, neutrophils may become primed in the process of diapedesis across the endothelial layers proximal to inflamed tissue engage various receptors. Interaction between fibrinogen and CR3 (CD11b/CD18) has been shown to induce rapid elevation of intracellular Ca^2+^, along with enhancement of CD66 and CD11b expression on the neutrophil cell surface, and induction of degranulation, suggesting mediation of neutrophil responses (19, 20). Fibrinogen from plasma adsorbs onto polystyrene surfaces (21). To investigate the effects of this interaction on CRIg expression by priming of neutrophils, we examined the effect of neutrophil interaction with plasma-coated 5 mL polystyrene FACS tubes. This did not cause any increases in CRIg expression (**Figure 3F**). While this needs to be studied more extensively, our findings suggest that the function of increased CRIg expression on neutrophils is likely to be predominantly dependent on inflammatory mediators.

### Effects of the Anti-Inflammatory Cytokine, IL-10

While some cytokines may activate neutrophils, others have been shown to depress their response, such as IL-10 (22). We thus examined the effects of the anti-inflammatory cytokine, IL-10. Neutrophils treated with IL-10 showed significant drop in basal expression of CRIg (**Figure 3G**). The cytokine also prevented the increase in expression caused by GM-CSF (**Figure 3G**).

### Role of PKC and p38 MAP Kinase

**Figure 4:**
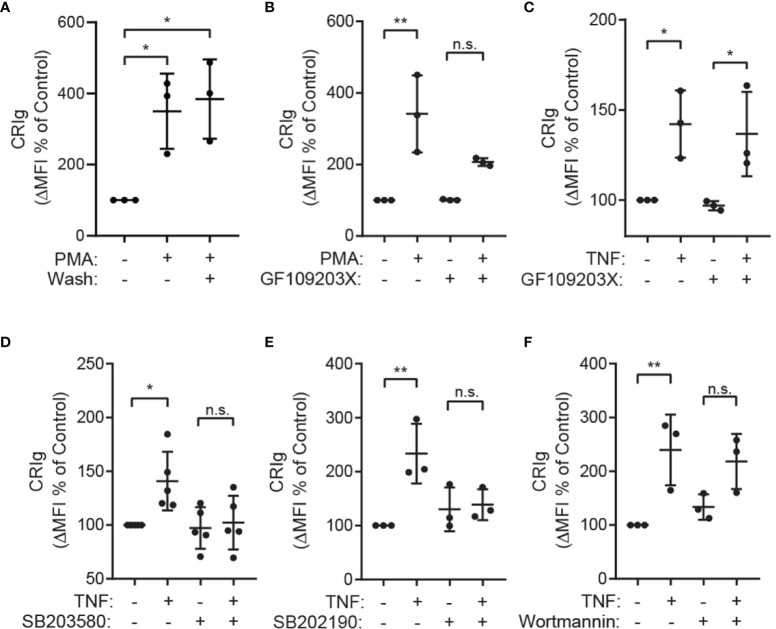
The role of PKC and p38 MAP kinase in up regulation of CRIg expression. **(A)** Shows Effect of washing the cells following priming. Neutrophils were treated with 100 nM of PMA for 20 min and then washed and re-incubated for 1h. After incubation the level of CRIg expression was measured by flow cytometry. **(B, C)** Surface CRIg expression induced by PMA or TNF measured on neutrophils pre-treated for 10 min with 500 nM of the PKC inhibitor, GF109203X. **(D, E)** Effects of p38 inhibitors. Surface CRIg induced by TNF on neutrophils pre-treated for 30 min with either 10 µM SB203580 or 20 µM SB202190. **(F)** Effects of PI3 kinase inhibitor, wortmannin. Surface CRIg induced by TNF on neutrophils pre-treated for 10 min with 100 nM wortmannin. Expression was assessed as described in **Figure 1** with the calculated ΔMFI expressed as a percentage increase over control treatment. Measurements were taken from distinct specimens and graphs show mean ± SD of these measurements, Sidak’s post-test compared between TNF/PMA treatment and control or treatment in the presence of inhibitor. Significance levels are indicated by asterisks: *P < 0.05, **P < 0.01, n.s., not significant.

The observation that PMA, a direct activator of PKC in intact cells (23, 24), causes an increase in CRIg surface expression by neutrophils (**Figures 1E, G**) suggests that PKC is involved in the upregulation of CRIg. The effects of PMA were long lasting and were not affected by washing the cells after the treatment. This suggests that once the priming signal has been initiated no further presence of this stimulation is required to maintain the increased CRIg expression (**Figure 4A**). Not surprisingly, treating neutrophils with the PKC pharmacological inhibitor, GF109203X, prevents the PMA-induced expression of CRIg (**Figure 4B**). In contrast to neutrophils, expression in macrophages has previously been found to be decreased by PMA (10, 25). In comparison, with TNF, the inhibitor has no effect on the TNF-induced expression of this complement receptor in neutrophils (**Figure 4C**), consistent with findings that TNF does not activate PKC in neutrophils (26, 27).

The TNF-induced CRIg expression in neutrophils is dependent on p38 MAP kinase, demonstrated by the ability of the well-characterized p38 inhibitors SB203580 and SB202190 to abrogate this response (**Figures 4D, E**) (16). Other MAP kinases such as ERK1/2 and JNK are unlikely to be involved as TNF does not activate these in neutrophils (16). Further investigations showed that the TNF effect is not dependent on phosphoinositide 3-kinase (PI3K) activation, as this activation was not inhibited by wortmannin (**Figure 4F**).

### Role of Intracellular Ca^2+^, Rac, Rab27a and ARPC1B

**Figure 5:**
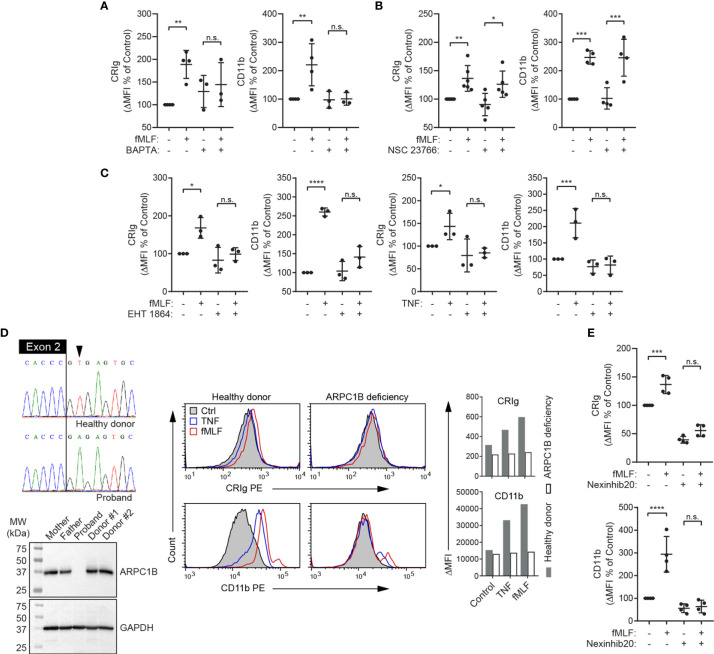
Expression of CRIg on the neutrophil surface is dependent on intracellular calcium, Rac, ARPC1B and Rab27a. **(A–C)**, Surface CRIg and CD11b expression was measured on neutrophils challenged with 10^-6^ M fMLF, following pre-treatment with either 25 μM of Ca^2+^ chelator, BAPTA-AM for 30 min, 50 μM of the Rac-1 inhibitor, NSC 23766 for 1 h, or 100 μM of the Rac-1/Rac-2 inhibitor, EHT 1864, for 30 min. The effect of EHT 1864 was also examined on TNF (10^3^ U/mL)-induced CRIg and CD11b expression. **(D)** Sequence electropherograms of the end of *ARPC1B* exon 2 and flanking intron in a healthy donor and a patient with a splice-site substitution (LRG_1188t1:c.64+2T>A). The resulting deficiency in ARPC1B is shown in the Western blot showing absence of the protein in neutrophil lysate compared to parental and other healthy donor neutrophils. Flow cytometric histograms present a lack of up-regulation of cell surface CRIg and CD11b in response to TNF is ARPC1B-deficient neutrophils. Graphs show mean ΔMFI of two experiments. **(E)** Surface CRIg and CD11b expression was measured on neutrophils challenged with 10^-6^ M fMLF, following pre-treatment with 20 μM of Rab27a inhibitor, Nexinhib20, for 1 h. Expression was assessed as described in **Figure 1** with either ΔMFI *per se* or as a percentage increase over control treatment. Measurements were taken from distinct specimens and graphs show mean ± SD of these measurements. One-way ANOVA with Sidak’s post-test compared between fMLF/TNF treatment and control or fMLF/TNF in the presence of inhibitor. Significance levels are indicated by asterisks: *P < 0.05, **P < 0.01, ***P < 0.001, ****P < 0.0001, n.s., not significant.

The above observations (**Figures 2**, **3**) imply that CRIg is stored intracellularly and mobilized to the cell surface during stimulation, presumably *via* the exocytotic pathway, as per macrophages (1). Neutrophil degranulation *via* exocytosis is known to require an increase in intracellular Ca^2+^ (28) and involves actin remodeling and microtubule assembly (29). We therefore investigated whether the increase in cell-surface CRIg expression requires intracellular Ca^2+^ and the function of the actin cytoskeleton. Previous studies in neutrophils have demonstrated that extracellular Ca^2+^ was minimally required for exocytosis whereas Ca^2+^ release from intracellular stores was critical for this function (reviewed in (28)). We therefore used the cell permeable Ca^2+^-selective chelator, BAPTA, in our investigations. When neutrophils were treated with BAPTA, there was no significant increase in expression of either CRIg or CD11b induced by fMLF (**Figure 5A**), implying a requirement for intracellular Ca^2+^.

To further investigate the role of the actin cytoskeleton, we examined the effects of blocking the function of rac, a small GTPase that regulates actin polymerization and neutrophil functions (30, 31). Three highly homologous forms of rac proteins (rac1, rac2 and rac3), are expressed in mammalian cells. Of these, rac1 and rac2 are expressed in neutrophils, and there is no evidence for the presence of rac3 in myeloid cells (32). Human neutrophils express predominantly rac2 (~97%) and a small amount of rac1 (33). Activation of these small GTPases in human neutrophils in response to fMLF has been demonstrated (34). We found that treating neutrophils with the rac1 inhibitor, NSC 23766, doesn’t alter either CRIg or CD11b expression in response to fMLF (**Figure 5B**). Additionally, EHT 1864, a rac inhibitor with selectivity for rac1 and rac2 over rac3 (35), suppresses the stimulatory effect of fMLF and TNF on CRIg and CD11b expression (**Figure 5C**), highlighting the importance of rac2.

To support a role for rac2 and actin filament reorganization in the induction of CRIg surface expression, we utilized neutrophils from a patient deficient in the expression of actin related protein 2/3 complex subunit 1B (ARPC1B) (**Figure 5D**), also known as Arc-p41, the only ARPC1 isoform expressed in hematopoietic cells (36, 37). Arp2/3 is a downstream effector of rac2 (38) and is required for actin filament branching (39). Our data demonstrate that ARPC1B deficient neutrophils, while showing normal basal expression of CRIg and CD11b did not display an increase in expression of these receptors in response to TNF and fMLF (**Figure 5D**).

Membrane-membrane docking and fusion during exocytosis requires intracellular Ca^2+^, as well as other effectors, including the small GTPase, Rab27a (40, 41). We therefore investigated whether Rab27a is required for the surface expression of CRIg. Nexinhib20, a specific Rab27a inhibitor, causes inhibition of CRIg expression (**Figure 5E**). Taken together, the above data support the contention that CRIg is recruited to the cell membrane from an intracellular compartment by the exocytotic pathway that requires a rise in intracellular Ca^2+^ concentration, reorganization of the actin cytoskeleton and a membrane fusion step that is regulated by Rab27a. Depending on the stimulus, this process also requires either PKC and/or p38, depending on the activating agonist.

## Discussion

The data demonstrate that neutrophils express CRIg mRNA and protein. Only one transcript of CRIg was observed in neutrophils and by western blot analysis the commercial antibody clone 6H8 detected one protein of molecular weight 35 kDa. This contrasted with MDM which showed in addition, a longer form, 50 kDa protein, as previously reported (1). A significant finding from our work was that expression on the cell surface required activation with inflammatory mediators and this then led to the activation of neutrophil functional responses through CRIg engagement with the anti-CRIg monoclonal antibody. CRIg expression by neutrophils may have been previously missed as in our study, we found that expression on the cell surface is poor, unless the neutrophils have been stimulated with inflammatory mediators or agonists. A wide range of mediators were able to cause an increase in CRIg expression and included both endogenously and exogenously generated mediators. Here we have demonstrated that fMLF, LPS and the cytokines TNF and GM-CSF induce expression of CRIg on the neutrophil surface. This effect extended to the lipid mediator LTB4 generated through the lipoxygenase pathway of the metabolism of arachidonate. The strong neutrophil activator PMA was very effective in inducing CRIg expression. The signals leading to increased CRIg expression were long-lived and did not require the continuous presence of the mediator, demonstrated for GM-CSF and PMA. Thus washing the cells free of these agonists did not reduce the increase in CRIg expression. While these agonists effectively increased CRIg expression it is unlikely that their priming in the process of diapedesis plays a role as there was no increase in CRIg expression when neutrophils were bound to plasma coated tubes, where fibrinogen in the plasma interacts with αMB2 on the neutrophils.

Of major interest was the finding that the expression of CRIg could be decreased by treating neutrophils with the anti-inflammatory cytokine IL-10. Thus, this cytokine caused a decrease in basal CRIg expression. When added to neutrophils together with GM-CSF, it affected the ability of this cytokine to increase CRIg expression. This is consistent with previous studies which showed a decrease in ability to induce a respiratory burst by GM-CSF if the neutrophils had been pretreated with IL-10 (22). While the IL-10 effects can be considered as causing a decreased ability to fight infections, it is likely to play a protective role in autoimmunity and tissue damage.

CRIg is a promoter of phagocytosis, and as it binds to the complement component C3b which is a precursor to iC3b, it is able to act more rapidly than the other complement receptors, such as CR3 (1, 42). Thus, for neutrophils, the ability to upregulate a receptor with such efficient phagocytic capability would be highly beneficial in the cell’s anti-microbial function. While Kupffer cells of the murine liver show high expression of CRIg (1), this expression is not present in human liver (9). Hence, here the infiltrating neutrophils seen in infection of the liver may play a critical role as anti-microbial cells since they would express CRIg on their surface. TNF primes neutrophils to kill bacteria and parasites in a complement-dependent manner (12, 43) and hence the increase in expression of CRIg is likely to contribute to this priming role, especially as CRIg is able to engage complement-opsonized bacteria before CR3 (1, 42). Our data demonstrate that the increased killing of *S. aureus* induced by TNF could be abrogated by blocking the interaction of bacteria with an anti-CRIg antibody. Furthermore, it was evident that upon increasing CRIg expression in TNF primed neutrophils, engaging the receptor with the anti-CRIg antibody induced a respiratory burst involving the NADPH oxidase and the generation of oxygen reactive species in association with increased activation of p38 MAP Kinase. Thus, the findings demonstrate that CRIg not only promotes neutrophil bacterial phagocytosis but also promotes their killing. The anti-bacterial properties of CRIg expression on neutrophils was further demonstrated by examining NET formation by human neutrophils. While GM-CSF was not able to induce NETs, it primed for NET formation in neutrophils challenged with anti-CRIg antibody. Previously we have shown that these agonists such as TNF also cause an increase in activation markers such as CR3 and FcγR (12, 43) and their relationship to the CRIg-promoted bacterial killing will need further investigation.

Studies into the mechanisms of the agonist-induced CRIg expression on neutrophils suggest a role for PKC, p38 MAP kinase, ARPC1B, rac2, Rab27a. The increase in CRIg surface expression induced by PMA, an activator of PKC, was inhibited by the pharmacological inhibitor, GF109203X. In comparison, the effects of TNF, which does not activate PKC (26, 27) were not inhibited, suggesting that CRIg expression can be induced *via* a PKC-dependent and PKC independent pathways. The response to TNF was dependent on p38, shown by inhibition by two of the p38 MAP kinase inhibitors, SB203580 and SB202190. Inhibition of Rab27a successfully inhibited surface upregulation of CRIg in response to fMLF stimulation. Rab27a is known to be responsible for regulating the docking and fusion with the plasma membrane during exocytosis of granule constituents (41).

A key observation emanating from the data is that CRIg expression is regulated differently in neutrophils compared to macrophages. While TNF, PMA and LPS depress expression in macrophages (10, 11), they increase expression in neutrophils. Indeed, PKC activation in macrophages depresses CRIg expression (25), but increases expression in neutrophils. Similarly, the activation of p38 is not required for macrophage CRIg expression (10).

Our observations that neutrophils express CRIg, has broad implications in terms of the potential use of CRIg as a therapeutic agent or target. Similar to other B7 family ligands, CRIg expression levels have been shown to be elevated in a range of cancers (44). As a result, it has been suggested that CRIg may be a potential target for inhibiting antibodies (similar in action to blocking antibodies against the checkpoint molecules PD-1 and PD-L1). However, if CRIg is naturally expressed in blood cells as well as the tissues and is an important player in neutrophil-mediated clearance of opsonized pathogens, then blocking CRIg may severely immunocompromise patients. Therefore, the properties of CRIg as identified here will be important in considering future studies into the therapeutic efficacy of CRIg-targeting agents.

The findings established that neutrophils express functional CRIg on the cell surface when activated by inflammatory mediator. This places another perspective on the mechanisms of the inflammatory reaction in infection and immunity involving CRIg. It is tempting to speculate that tissue infiltrating neutrophils are armed with CRIg to participate in phagocytosis in bacterial and fungal infection. While the cells in an unstimulated state show little or no CRIg expression, with the generation of inflammatory mediators there is an increase on neutrophils, enabling this cell to efficiently participate in CRIg mediated phagocytosis of bacteria.

## Data Availability Statement

The original contributions presented in the study are included in the article/supplementary material. Further inquiries can be directed to the corresponding author.

## Ethics Statement

The studies involving human participants were reviewed and approved by Human Research Ethics Committee of the Women’s and Children’s Health Network and Southern Adelaide Clinical Human Research Ethics Committee. The patients/participants provided their written informed consent to participate in this study.

## Author Contributions

AS and AF were responsible for the initial draft of the manuscript. AF proposed and supervised the study and together with AS, KP, CH, and AQ, planned the experiments. In the main the experiments were carried out by AS and KP with a contribution by TP and NP. Authors PQ, AQ, TP, and AF were responsible for the clinical and laboratory work up of the patient with ARPC1B deficiency. AS, KP, and AQ were involved in data collation, statistics and presentation. AS, AF, CH, MW, and AQ were responsible for critically reading the manuscript. All authors contributed to the article and approved the submitted version.

## Funding

The work was supported by grants from the NHMRC of Australia and the Channel 7 Children’s Research Foundation.

## Conflict of Interest

The authors declare that the research was conducted in the absence of any commercial or financial relationships that could be construed as a potential conflict of interest.

## Publisher’s Note

All claims expressed in this article are solely those of the authors and do not necessarily represent those of their affiliated organizations, or those of the publisher, the editors and the reviewers. Any product that may be evaluated in this article, or claim that may be made by its manufacturer, is not guaranteed or endorsed by the publisher.
